# Neurophysiological and clinical biomarkers of secondary progressive multiple sclerosis: A cross-sectional study

**DOI:** 10.3389/fneur.2023.1138600

**Published:** 2023-03-16

**Authors:** Matteo Tartaglia, Marco Canevelli, Leonardo Malimpensa, Daniele Belvisi, Viola Baione, Gina Ferrazzano, Giorgio Leodori, Alfredo Berardelli, Antonella Conte

**Affiliations:** ^1^Department of Human Neurosciences, Sapienza University of Rome, Rome, Italy; ^2^Department of Neurophysiology, IRCCS Neuromed, Pozzilli, Italy

**Keywords:** multiple sclerosis, frailty, neurophysiology, disease progression, biomarkers, transcranial magnetic stimulation

## Abstract

Timely diagnosis of secondary progressive multiple sclerosis (SPMS) represents a clinical challenge. The Frailty Index, a quantitative frailty measure, and the Neurophysiological Index, a combined measure of sensorimotor cortex inhibitory mechanism parameters, have recently emerged as promising tools to support SPMS diagnosis. The aim of this study was to explore the possible relationship between these two indices in MS. MS participants underwent a clinical evaluation, Frailty Index administration, and neurophysiological assessment. Frailty and Neurophysiological Index scores were found to be higher in SPMS and correlated with each other, thus suggesting that they may capture similar SPMS-related pathophysiological mechanisms.

## 1. Introduction

Secondary progressive multiple sclerosis (SPMS) develops after a relapsing-remitting form of multiple sclerosis (RRMS). SPMS diagnosis is retrospectively based on worsening of the Expanded Disability Status Scale (EDSS) scores, without substantial changes in magnetic resonance imaging (MRI). There is evidence that a high EDSS score at MS diagnosis is a risk factor for RRMS-to-SPMS conversion (1). However, EDSS assessment is affected by significant inter-rater variability and a substantial frequency of rating errors depending on the examiner's experience (2).

The identification of possible objective markers of RRMS-to-SPMS conversion is therefore needed. A neurophysiological marker to identify SPMS has been recently proposed, which consists of an index derived from the objective assessment of a neurophysiological and a psychophysical variable. The first is short intracortical inhibition (SICI), which tests inhibitory interneuron excitability in the motor cortex. The second is the somatosensory temporal discrimination threshold (STDT), which tests inhibitory interneuron excitability in the primary sensory cortex (3, 4).

The neurophysiological index combining SICI and STDT also included age as a factor in the formula predicting SPMS. This observation is in line with previous evidence suggesting that chronological aging may play a role in the RRMS-to-SPMS transition (5). Besides chronological aging, however, also biological aging, as measured by Frailty Index (FI), seems to be associated with SPMS (6). The FI is a quantitative frailty indicator based on clinical and laboratory data that assesses an individual's global vulnerability to stressors and may represent a useful multidimensional tool to evaluate biological aging in MS. FI is able to discriminate SPMS from RRMS and has been proposed as a possible clinical marker for SPMS (6). Intriguingly, the association between frailty and SPMS is lost in the advanced phases of the disease (7), thus suggesting that frailty should be considered a factor implied in the conversion from RRMS to SPMS rather than a long-term feature of SPMS. From this perspective, FI could be considered a quantitative clinical marker of SPMS at its early stages.

Both the neurophysiological index and FI correlate with chronological aging in MS but it is unknown whether a relationship between these potential biomarkers for MS is present.

The aim of this study was to investigate the neurophysiological index and the FI comparing RRMS and SPMS patients and explore possible correlations between these two measures.

## 2. Materials and methods

### 2.1. Subjects

For this purpose, 19 patients with MS (13 RRMS, mean age 41.2 ± 9.0 years, median EDSS 1.0 [0–4.0]; 6 SPMS, mean age 53.3 ± 5.2 years, median EDSS 6.0 [2.5–7.5]) were enrolled at the Multiple Sclerosis Outpatient Clinic, Department of Human Neurosciences, Sapienza University of Rome. MS diagnosis was defined accordingly to the latest revised McDonald criteria (8), while disease course was identified based on Lublin definition (9). Inclusion criteria were age over 18 years, RRMS or SPMS diagnosis, absence of contraindications to TMS (such as epilepsy or head trauma). SPMS enrolled patients were not in an active phase as defined by Lublin et al. (9), while RRMS patients had to be free from relapses and from corticosteroid intake in the 30 days preceding the assessments. All study participants gave a written informed consent. The study was approved by the Ethical Committee of our Institution and was conducted according to the Declaration of Helsinki. All patients underwent clinical and neurophysiological examination.

### 2.2. Neurophysiological index

All neurophysiological assessments were performed in a random order while patients were comfortably sitting on an armchair. The neurophysiological index was calculated for each patient using the following formula obtained in a previous work (3):


$$\begin{array}{l} P\left(X=1\right)=\frac{e^{-5.95503+0.00056^{*}SICI{\left(\%\right)}^{*}Age+0.00073^{*}STDT^{*}Age}}{1+e^{-5.95503+0.00056^{*}SICI{\left(\%\right)}^{*}Age+0.00073^{*}STDT^{*}Age}}\; \end{array}$$

This score computes the probability that a patient (X) has to be assessed as SPMS (class 1) using three variables: age, SICI (%), and STDT.

#### 2.2.1. Short intracortical inhibition (SICI)

We delivered single and paired-pulses through a Magstim Bistim2 magnetic stimulator (The Magstim Company, Ltd., Whitland, South West Wales, UK) connected to a figure-of-eight coil. Motor evoked potentials (MEPs) from the first dorsal interosseous muscle were elicited delivering transcranic magnetic stimulation (TMS) to the contralateral M1 motor area. Coil was handled tangentially to the scalp, while tail was placed backward at 45° respect of the median line. Minimum single-pulse intensity able to elicit a 50 μV of amplitude MEP was defined as resting motor threshold (RMT). Consequently, conditioning stimulus intensity was set as 80% of RMT while TMS intensity able to evoke a 1 mV of average amplitude MEP was used as test stimulus. To assess SICI, a 3 ms interstimulus interval (ISI) between conditioning and test stimulus was used. SICI effects were then computed as the percentage ratio between the conditioned MEP amplitude and the test MEP amplitudes [SICI (%)].

#### 2.2.2. Somatosensory temporal discrimination threshold (STDT)

To test STDT we used procedures explained in previous studies (3, 10, 11). Briefly, pairs of square-wave electric stimuli were delivered with an increasing ISI of 10 ms starting from a couple of simultaneous stimuli. Electric stimulation was performed through a stimulator (Digitimer DS7AH) connected to AgCl electrodes placed on the volar face of the right index finger. Stimulation intensity was increased, starting from 2 mA, by 1 mA for each step to reach the minimum intensity at which patients perceived 10 out of 10 stimuli. STDT was defined as the first of three consecutive ISIs when patients could temporally discriminate the stimuli. During the experiment “catch trials” were performed to reduce persevering answers and to check patient's attention level.

### 2.3. Frailty index (FI)

The FI was assessed through 42 clinical and laboratory health items as previously described (6). During the outpatient visit, subjects were questioned about each item and a score of 1 was assigned if a deficit was present and of 0 if absent. The FI score was then computed for each participant as a ratio between the total number of deficits and the total number of items (*n* = 42).

### 2.4. Statistical analysis

Mann-Whitney U test for independent samples was used to evaluate differences in the neurophysiological and frailty indices between RRMS and SPMS patients. Spearman's correlation coefficient was used to investigate possible correlations between the FI and neurophysiological index. This analysis was adjusted for disease duration and sex. A case-wise diagnostic was used to detect outliers with standardized residual >±2 standard deviations. The case-wise diagnostic detected 1 outlier (case 9 in Table 1) in the relation between FI and neurophysiological index.

**Table 1:** Patients' demographic, neurophysiological and frailty characteristics.

**ID**	**Age (years)**	**Sex**	**Clinical phenotype**	**EDSS (score)**	**Disease duration (years)**	**Neurophysiological index (score)**	**Frailty index (score)**
1	49	F	RR	0	21	0.06	0.14
2	34	M	RR	0	1	0.31	0.07
3	51	F	RR	2	11	0.65	0.19
4	30	F	RR	0	5	0.05	0.05
5	30	F	RR	1	1	0.01	0.19
6	55	M	RR	1.5	28	0.21	0.26
7	52	F	RR	1.5	9	0.52	0.11
8	48	F	RR	1	6	0.11	0.14
9	38	F	RR	2	4	0.15	0.38
10	40	M	RR	4	13	0.07	0.07
11	38	M	RR	1	8	0.09	0.05
12	29	M	RR	1.5	6	0.02	0
13	42	F	RR	1	11	0.18	0.12
14	49	M	SP	3.5	26	0.78	0.19
15	52	M	SP	6	18	0.96	0.29
16	46	F	SP	7.5	21	0.94	0.33
17	59	F	SP	2.5	35	0.95	0.12
18	57	F	SP	7	22	0.99	0.33
19	57	F	SP	6	24	0.92	0.33
RRMS mean (SD)	41.2 (9.0)	F = 8	-	-	9.5 (7.7)	0.19 (0.19)	0.14 (0.10)
SPMS mean (SD)	53.3 (5.2)	F = 4	-	-	24.3 (5.9)	0.92 (0.07)	0.27 (0.09)

EDSS, Expanded disability status scale; RRMS, Relapsing-remitting multiple sclerosis; SPMS, Secondary progressive multiple sclerosis.

## 3. Results

As expected, patients with SPMS were older than patients with RRMS (*p* = 0.009) and had higher EDSS values (*p* < 0.001). Consistent with previous studies, the neurophysiological index differed between RRMS (0.19 ± 0.19) and SPMS (0.92 ± 0.07) (*p* < 0.001). The FI was also significantly higher in patients with SPMS (0.27 ± 0.09) than in patients with RRMS (0.14 ± 0.10) (*p* = 0.02). Both FI (ρ = 0.6; *p* = 0.008) and neurophysiological index (ρ = 0.7; *p* = 0.001) correlated with EDSS.

**Figure 1:**
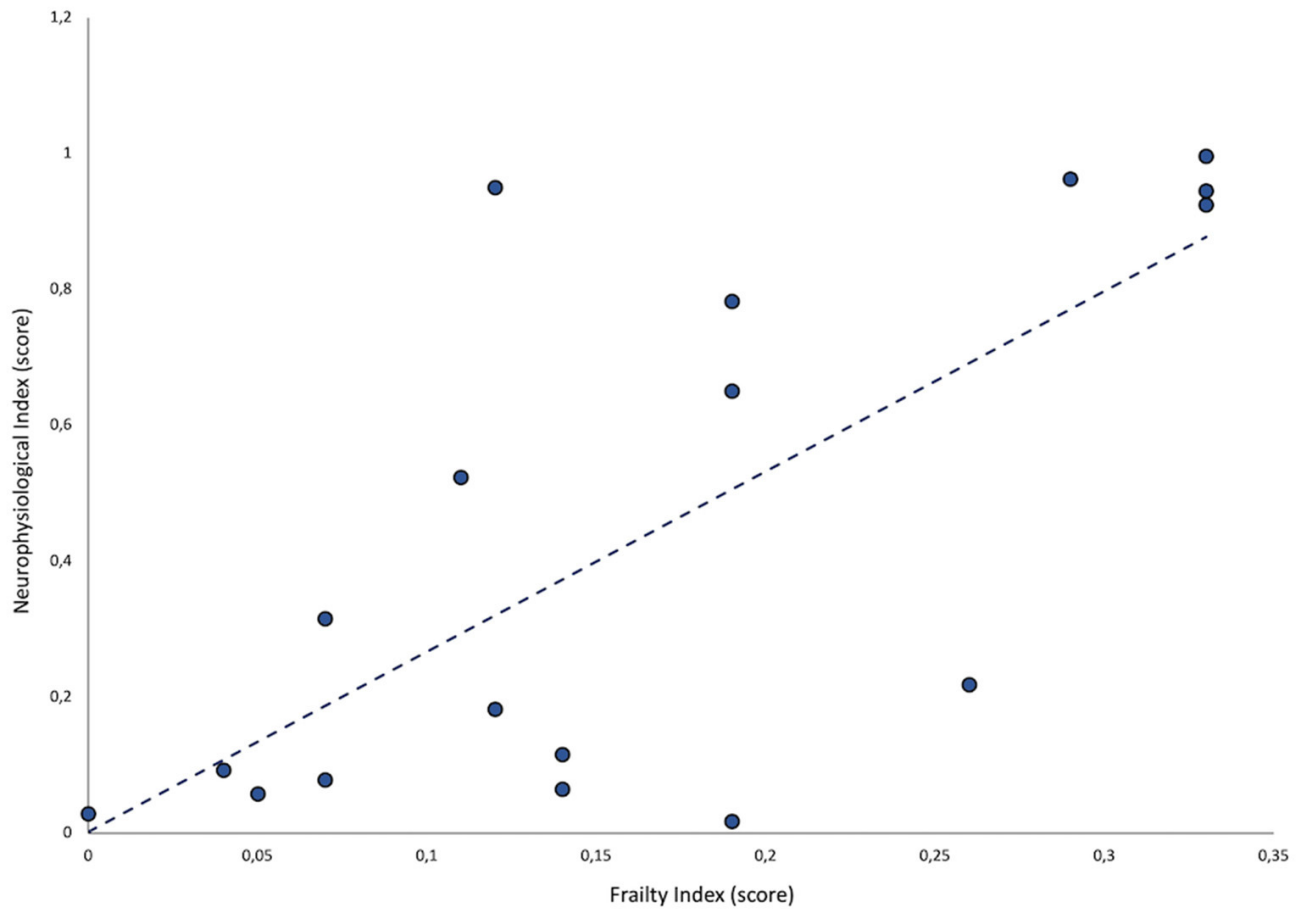
Correlation between neurophysiological index (NI) score and frailty index (FI) score.

A statistically significant, positive correlation between FI and neurophysiological index values was observed (ρ = 0.5; *p* = 0.03) (Figure 1).

## 4. Discussion

The positive correlation between the neurophysiological and frailty indices in patients with MS suggests that both reflect similar pathophysiological mechanisms involved in the progression to SPMS. Higher frailty levels and abnormalities in the considered neurophysiological measures may capture those neurodegenerative processes that underlie the progressive course of the disease. Abnormalities in both neurophysiological and frailty indices may depend on the involvement of gray matter (3, 6). This conclusion is also supported by MRI evidence of higher gray matter loss in patients with SPMS as compared to those with RRMS (12).

The correlation between FI and neurophysiological index also provides a more comprehensive understanding of the role of these candidate biomarkers in MS. For instance, the present observation that FI and neurophysiological index correlate in MS suggests that the age-related accumulation of health/biological deficits (as expressed by FI) is associated with neurophysiological changes that intervene at cortical level, thus reflecting neurodegenerative rather neuroinflammatory processes. This provides a neurobiological substrate to the previously documented role of frailty on MS clinical expression.

The cross-sectional design constitutes the main limitation of the present study. The correlation that emerged between the neurophysiological index and FI should be interpreted with caution, and follow-up longitudinal investigations are needed to clarify their mutual relationship.

To conclude, we suggest the assessment of neurophysiological and frailty indices as objective markers in identifying patients at risk of disease progression. Future longitudinal investigations in naïve patients with MS are needed to demonstrate the predictive value of the frailty and neurophysiological indices in identifying RRMS-to-SPMS transition.

## Data availability statement

The raw data supporting the conclusions of this article will be made available by the authors, without undue reservation.

## Ethics statement

The studies involving human participants were reviewed and approved by Sapienza University of Rome Ethics Committee. The patients/participants provided their written informed consent to participate in this study.

## Author contributions

Conceptualization: AC, DB, and MC. Investigation: MT, LM, VB, GF, and GL. Writing—original draft: MT, MC, and DB. Writing—review and editing: MC, DB, AB, and AC. All authors approved the final version.
